# High Concentrations of Sodium Chloride Improve Microbicidal Activity of Ibuprofen against Common Cystic Fibrosis Pathogens

**DOI:** 10.3390/ph11020047

**Published:** 2018-05-17

**Authors:** Adrián J. Muñoz, Roxana V. Alasino, Ariel G. Garro, Valeria Heredia, Néstor H. García, David C. Cremonezzi, Dante M. Beltramo

**Affiliations:** 1 Centro de Excelencia en Productos y Procesos de Córdoba, Gobierno de la Provincia de Córdoba, Pabellón CEPROCOR, Santa María de Punilla, Córdoba CP 5164, Argentina; adrianmunoz30@yahoo.com.ar (A.J.M.); garroariel@hotmail.com (A.G.G.); valeriaheredia90@hotmail.com (V.H.); 2 Consejo Nacional de Investigaciones Científicas y Técnicas, CONICET, Godoy Cruz 2290, C1425FQB CABA, Argentina; nestorhgarcia11@gmail.com; 3 Instituto de Investigaciones en Ciencias de la Salud—FCM (INICSA-CONICET), Córdoba C 5000, Argentina; 4 Departamento de Salud y Educación Cátedra de Patología, Universidad Nacional de La Rioja, La Rioja C 5300, Argentina; dccj35@yahoo.com.ar; 5 Cátedra de Patología, Universidad Nacional de Córdoba, Córdoba C 5000, Argentina; 6 Cátedra de Biotecnología, Facultad de Ciencias Químicas, Universidad Católica de Córdoba, Córdoba C 5000, Argentina

**Keywords:** ibuprofen, *P. aeruginosa*, cystic fibrosis, bactericide activity, synergy

## Abstract

Ibuprofen (IBU-H), a widely used anti-inflammatory, also shows a marked antimicrobial effect against several bacterial species, including those involved in cystic fibrosis such as *Pseudomona aeruginosa*, methicillin resistant *Staphylococcus aureus* and *Burkholderia cepacia* complex. Additionally, our results show significant synergy between water soluble Na-ibuprofen (IBU-Na) and ionic strength. Salt concentrations above 0.5 M modify the zeta potential promoting the action of Na-IBU; thus, with 1 M sodium chloride, IBU-Na is ten times more efficient than in the absence of ionic strength, and the minimum effective contact time is reduced from hours to minutes. In short time periods, where neither IBU-Na nor controls with 1 M NaCl show activity, the combination of both leads to a reduction in the bacterial load. We also analyzed whether the changes caused by salt on the bacterial membrane also promoted the activity of other microbicide compounds used in cystic fibrosis like gentamicin, tobramycin and phosphomycin. The results show that the presence of ionic strength only enhanced the bactericidal activity of the amphipathic molecule of IBU-Na. In this respect, the effect of saline concentration was also reflected in the surface properties of IBU-Na, where, in addition to the clear differences observed between 145 mM and 1 M, singular behaviors were also found, different in each condition. The combination of anti-inflammatory activity and this improved bactericidal effect of Na-IBU in hypertonic solution provides a new alternative for the treatment of respiratory infections of fibrotic patients based on known and widely used compounds.

## 1. Introduction

Since the discovery of penicillin in the 1920s followed by that of sulfonamides in the 1930s, many other antibiotics have been discovered, developed and commercially produced. Unfortunately, several factors contribute to the selection of resistant microbial strains which determine the fact that more pathogens become resistant to many chemotherapeutic agents [1,2,3,4,5,6]. Thus, the development of new drugs with novel mechanisms of action has become one of the major priorities in human health [7].

Although some innovative compounds are understudy, only a few new classes of antibiotics have been developed since the late 1980s. Much of the research has focused on the chemical modification of existing drugs to improve their power and to overcome antibiotic resistance mechanisms [8,9,10,11]. In recent times, compounds currently used for the treatment of non-infectious diseases as potential antimicrobial alternatives have also been reviewed [12,13]. These compounds exert their antimicrobial activity through different mechanisms, affecting membrane, altering metabolism and intercalating DNA through the suppression of adhesion, among others [13].One example is Ibuprofen (IBU), a nonsteroidal anti-inflammatory drug (NSAID) widely used for relief of pain, fever and inflammation, which has demonstrated microbicidal activity [14,15]. 

In this sense, the antibacterial and antifungal activity of IBU was reported for the first time by Hersh et al.in 1991 and Sanyalet al.in 1993, respectively [16,17]. These works promoted new studies describing the antimicrobial effect of IBU. However, the use of this compound is still confined to its anti-inflammatory activity. The compound has also serious drawbacks for administration by injection. 

In this paper we study the antimicrobial activity of IBU against Gram (+) and (−) species, particularly against those pathogens involved in cystic fibrosis (CF), such as *P. aeruginosa*, methicillin resistant *S. aureus* and *B. cepacia*. The CF is a genetic disorder caused by a defective gene that mostly affects the lungs, but also the pancreas, liver, kidneys and intestine. CF patients have abnormally thick, sticky mucus that accumulates in the airways of the lungs and pancreas leading to life-threatening lung infections and serious digestive problems. 

Although IBU has already been considered for the treatment of infections in fibrotic patients and its oral administration improves the respiratory illness of these patients, in the present work we specifically consider the effect of ionic strength on the activity of IBU-Na. Over the past years, many studies have described the benefits of an inhalation of hypertonic saline (HTS) in the treatment of patients with or without fibrotic lung disease [18,19,20]. These reports show that the use of HTS improves significantly the mucus clearance, thereby enhancing lung function. Another mechanism closely related to these diseases, especially in cystic fibrosis patients, is connected to the inflammation occurring in the airways as a result of the combination of a deregulated immune response and an innate infection. In this regard, some reports indicate that hyperosmolar inhalation therapy moderates neutrophil-mediated cytotoxicity, limiting some of the mediators of inflammation processes such as cell adhesion, production of reactive oxygen species and release of the protease. 

We also analyze the correlation between our in vitro and in vivo results and the behavior of the compound in air-buffer interfaces and its interaction with monomolecular layers of lipids in different conditions of NaCl concentrations. 

## 2. Materials and Methods 

### 2.1. Materials 

#### 2.1.1. Reagents

IBU was obtained from Todo Droga Chemical Co. (Córdoba, Argentina). Stock solutions of 2 M IBU-H (ibuprofen in protonated form) was prepared dissolving IBU-H in 96% ethanol (EtOH98%, Cicarelli, Córdoba, Argentina). In all conditions tested the final ethanol concentration of IBU-H solutions was 5%, a condition where bacterial viability was not affected. For 200 mM IBU-Na working solution, IBU-H was dissolved in sterile distilled water adding NaOH 2 N solution to obtain IBU-Na pH 7.8. 

Antibiotics: Tobramycin (Tobra) was supplied lyophilized by Química Luar Pharmaceutical Co. (Córdoba, Argentina) and resuspended with distilled water to obtain working solutions of 46.7 mg/mL (0.1 M); Phosphomycin (Phospho) was supplied lyophilized by Química Luar Pharmaceutical Co. and resuspended with distilled water to obtain working solutions of 13.80 mg/mL (0.1 M). Gentamicin (Genta) was supplied by Richet Lab.(Buenos Aires, Argentina) in vials of 2 mL injection solution containing 80 mg of Genta (as sulphate), and used directly as working 0.06 M solutions.

#### 2.1.2. Microorganisms 

Microorganisms tested in this study were obtained from commercial strains of *P. aeruginosa* ATCC 23522 and *S. aureus* ATCC 6538 (KWIK-STIK Microbiologics, Manassas, VA, USA). An isolate from a CF hospitalized patient, grown in Burkholderia Cepacia Selective Agar (BCSA) medium, where only these pathogens grow, produced a shift from orange to red via biochemical typing tests: xylose (+), mobility (+), lactose 10% (+) and lysine (+) and API 20NE Kit (Bio-Merieux S.A., Marcy l’ Etoile, France) for phenotypic identification.

These microorganisms were grown at 35 ± 2 °C in tubes with 10 mL of sterilized BHI-Brain Heart Infusion (BK 015HA, Biokar, 93692 PANTIN CEDEX, France) for 24 h. In subsequent experiments, bacterial suspensions were washed with sterile water and adjusted to match 0.5 McFarland turbidity standards. Suspensions were stored for up to 3 weeks at a temperature not exceeding 8 °C. 

### 2.2. Methods 

#### 2.2.1. Microdilution Antibacterial Test for Minimum Inhibitory Concentration (MIC) 

The method consists of establishing a gradient of antimicrobial concentration as a means of determining susceptibility. MIC values were then determined by a microdilution method [21] with modifications; the viable colony count was performed following a pour plate count method (PPC) [22] with Muller Hinton (MH) agar in the presence of 1% 2,3,5-triphenyl-2*H* tetrazolium chloride (TTC) solution as a redox indicator of cellular respiration and incubated during 24–48 h at 35 ± 2 °C. 

#### 2.2.2. Effect of IBU on Bacterial Growth 

In vitro studies on the activity of IBU-Na and IBU-H were performed incubating bacterial suspensions in PBS with increasing concentrations (1, 5, 10, 25, 50 and 100 mM) of each IBU by different time periods at 35 ± 2 °C. After completing the incubation time, the count of viable organisms was performed using the PPC method. Data logs of colonies forming units (cfu) per milliliter were recorded on a graph as a function of drug concentration, showing MIC as the lowest concentration which inhibits microbial growth. The experiment was repeated three times for statistical analysis in this study. 

In order to evaluate the effects of pH on the activity of IBU-Na, suspensions of *P. aeruginosa* were also placed in PBS buffer at pH: 6.8, 7.3 and 7.8, with 50 mM IBU-Na at 35 ± 2 °C for 4 h. To determine the minimum exposure time in the presence of ionic strength to achieve MIC, suspensions of *P. aeruginosa* were incubated with IBU-Na (5, 10, 25, 50 and 100 mM) in PBS buffer of high ionic strength (1 M NaCl) and its viability was assessed after 10 min. 

Finally, to determine the effect of antibiotics in the presence of ionic strength to achieve MIC, suspensions of *P. aeruginosa* were incubated with Tobra, Phospho and Genta (0.1, 0.25, 0.5, 1, 2, and 5 mM) in PBS buffer of high ionic strength (1 M NaCl) and their viability was analyzed after 10 min.

#### 2.2.3. Langmuir Film Balance Experiments

Compression isotherms and ibuprofen adsorption at the air–water interface or penetration studies were carried out in a small custom-made PTFE trough (15 mL). Surface pressure was measured by means of Pt-plate. The whole system was enclosed in an acrylic box surrounded. Films were compressed at 3 Å^2^·molecule^−1^·min^−1^; two-fold reduction of the compression rate caused no changes in isotherms. Values are the average of three independent experiments.

Ibu-Na stock solution was prepared at a concentration of 800 mM in bidistilled water and stock solutions of bacterial phospholipids were prepared by dispersing in Cl:MeOH (2:1) to a final concentration of 2 mM. The subphase solution used for Langmuir monolayer experiments was 100 mM Buffer TRIS-HCl pH 7.6 with 145 mM or 1 M NaCl.

#### 2.2.4. Preclinical Toxicological Study 

Considering that the inhalation route has not yet been approved for the administration of IBU, an acute animal toxicity study was conducted to evaluate the local effect of respiratory IBU-Na in the lungs. The study was carried out in Wistar rats of 250–300 g and the experiments were conducted in accordance with the guidelines set forth by the National Institutes of Health (NIH) Guide for the Care and Use of Laboratory Animals. All the procedures were approved under code number RF1258/2017by the Institutional Committee for the Care and Use of Laboratory Animals at the School of Medical Sciences (Universidad Nacional de Córdoba, Argentina). 

Two increasing doses (25 and 50 mM) of IBU-Na were inhaled by A and B groups of animals (*n* = 8) using an animal inhalation chamber. Animals were nebulized 1 h a day for four months and monitored daily with respect to general health, food intake, water consumption and any morphological changes in cardio-respiratory system (short/fast breathing, restlessness, sluggishness). At the end of the study, animals were sacrificed and any histopathological changes in lungs and other vital organs of test animals were compared with those of controls. Groups C and D were treated in a similar way to A and B; yet, after 4 months in each case, [comma] they were left for 15 more days without any treatment and then sacrificed. 

## 3. Results 

### 3.1. Determination of Minimum Inhibitory Concentration (MIC) of Ibuprofen 

**Figure 1:**
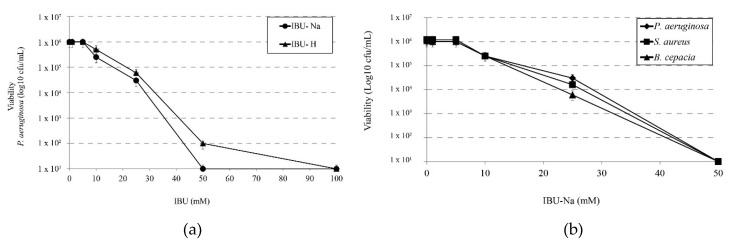
Minimum inhibitory concentration of IBU. (**a**) Bactericidal effect of increasing concentrations of IBU-Na and IBU-H (1, 5, 10, 25, 50, 100 mM) on the viability of *P. aeruginosa* at 1 × 10^6^ cfu/mL after incubation for 24 h at 35 ± 2 °C. (**b**) Susceptibility of *S. aureus*, *B. cepacia* and *P. aeruginosa* at initial concentration of 1 × 10^6^ cfu/mL to increasing concentrations of IBU-Na (1, 5, 10, 25, 50 mM) incubated at 35 ± 2 °C for 24 h.

We determined the minimum effective concentration of IBU on *P. aeruginosa* and compared the effect of salt (IBU-Na) versus the effect of its acid form (IBU-H). For this purpose, we tested increasing concentrations of IBU in both forms against a 1 × 10^6^ cfu/mL suspension of *P. aeruginosa*. The results in Figure 1a shows that IBU-Na presents lower MIC values (50 mM) than those of IBU-H (100 mM).

Accordingly, we evaluated the effect of IBU-Na against other three bacterial species of particular interest in CF such as *P. aeruginosa*, *S. aureus* and *B. cepacia*. Figure 1b shows that IBU-Na appears to be effective at the same concentrations against the three strains studied. This effect of IBU-Na was observed in a range of pH between 6.8 and 7.6 (data not shown).

### 3.2. Bactericidal Effect of IBU-Na as a Function of Time

**Figure 2:**
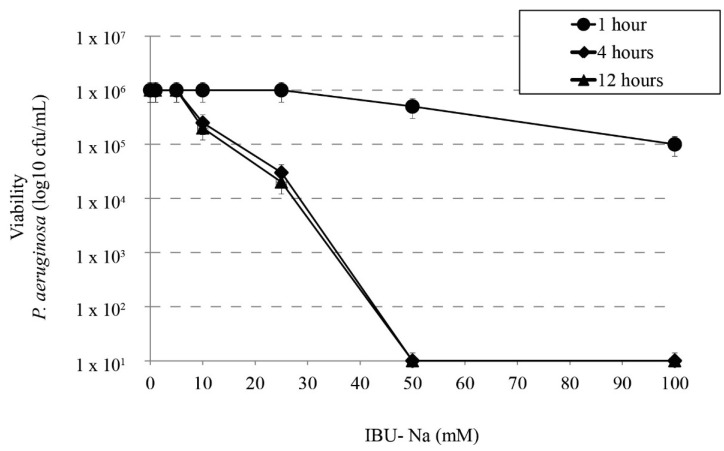
Effect of incubation time on bactericidal activity of IBU. Bactericidal effect of IBU-Na between 1 to 100 mM concentration at different incubation times (1, 4 and 12 h) on *P. aeruginosa* inoculum (1 × 10^6^ cfu/mL).

We studied the bactericidal effect of IBU-Na in relation to incubation time with *P. aeruginosa*, *S. aureus*, *B. cepacia*. Figure 2 shows that at least 4 h of incubation are required to observe bactericidal effect; at shorter times [1 h], no IBU-Na effect was found in the concentrations tested. Similar results were observed for *S. aureus*, *B. cepacia* (data not shown).

### 3.3. IBU-Na Activity as a Function of Initial Inoculum Title

**Figure 3:**
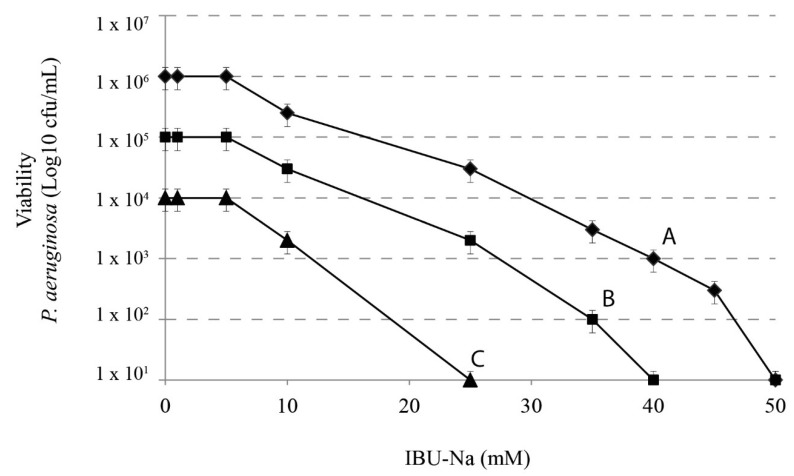
IBU-Na activity as a function of different initial inoculum titres. Effects of IBU-Na (1, 5, 10, 25 and 50 mM) were tested at three different initial inoculum densities referred to as Population A ♦-♦ (1 × 10^6^ cfu/mL), B ■-■ (1 × 10^5^ cfu/mL) and C ●-● (1 × 10^4^ cfu/mL), obtained from decimal serial dilutions of *P. aeruginosa* 1 × 10^7^ cfu/mL. The incubation was performed for 4 h at 37 °C.

We assessed the effect of IBU-Na according to the initial title of the bacterial inoculums. We used 10-fold serial dilutions of 1 × 10^6^ cfu/mL *P. aeruginosa* with concentrations of IBU-Na from 1 to 100 mM incubated for 4 h at 37 °C. Figure 3 shows that, when the initial concentration of *P. aeruginosa* increases from 10^4^ to 10^6^ cfu/mL, concentrations of IBU-Na from 1 to 5 mM show no bactericidal effect, even after 24 h of incubation (data not shown). The minimum effective concentration of IBU-Na against *P. aeruginosa* was around 10 mM; above 50 mM, IBU-Na completely eliminated inoculums of at least 1 × 10^6^ cfu/mL. Similar results were obtained for *S. aureus*, *B. cepacia* (data not shown).

### 3.4. Effect of Ionic Strength on the Bactericidal Effect of IBU-Na

**Figure 4:**
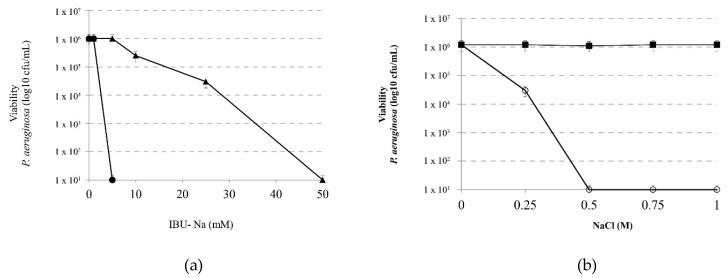
Effect of ionic strength on the bactericidal effect of IBU. (**a**) Viability of *P. aeruginosa* incubated with IBU-Na (1, 5, 10, 25 and 50 mM) in the absence (▲) and presence (⚫)of 1 M NaCl 4 h at 35 ± 2 °C. (**b**) Effect of salt concentration on the bactericidal effect of IBU-Na. Viability of a suspension of *P. aeruginosa* 1 × 10^6^ cfu/mL incubated for 60 min at 35 ± 2 °C with 10 mM IBU (○-○) as a function of salt concentration present in the medium. A control of NaCl effect without IBU (■-■) was assayed in similar conditions.

In order to analyze the effect of ionic strength on the IBU-Na bactericidal capacity, we evaluated the effect of increasing concentrations of IBU-Na (5, 10, 25, 50 and 100 mM) on *P. aeruginosa* (1 × 10^6^ cfu/mL) in the presence of 1 M NaCl for 4 h at 35 ± 2 °C. Figure 4a shows that concentrations as low as 5 mM of IBU-Na, normally with no effect on *P. aeruginosa*, reduced at least 6 log bacterial viability. Controls of *P. aeruginosa* incubated with 1 M NaCl showed that the presence of ionic strength does not affect significantly the viability of the bacteria between 0 to 4 h of incubation (data not shown).

Moreover, in order to determine the minimum amount of NaCl required by modifying the activity of IBU-Na, we evaluated the effect of 10 mM IBU-Na with increasing concentrations of NaCl on *P. aeruginosa*. Figure 4b shows that low concentrations of NaCl, between 10 and 250 mM, do not alter the antimicrobial activity of IBU-Na. However, at concentrations above 500 mM, a significant increase was found in the bactericidal activity of IBU-Na.

We also evaluated the effect of ionic strength on the kinetics of antimicrobial activity of IBU-Na. 1 M NaCl, in addition to reducing the MIC of 50 to 5 mM, also significantly decreased the time required to perform its antibacterial effect, which fell from 4 h to 5 min (data not shown). 

It is important to note that the effect of ionic strength was observed only as salt was present in the medium during incubation. Removing salt content of the medium by centrifugation and resuspension of bacteria in culture medium has shown that the values of the effect of IBU-Na return to those found initially. 

### 3.5. Effect of Ionic Strength on the Effect of Other Microbicides

**Figure 5:**
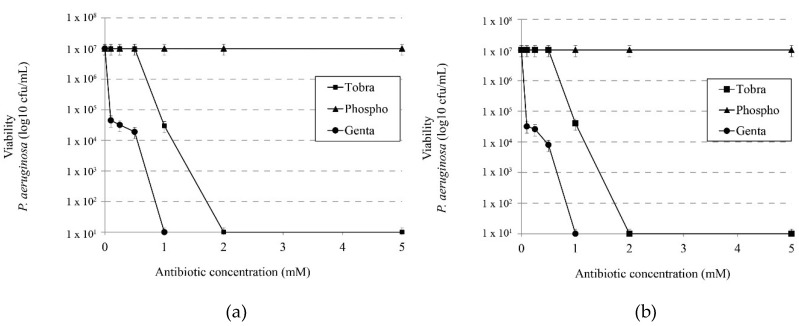
Effect of ionic strength on the microbicidal activity of different antibiotics. Effects of tobramycin, phosphomicine and gentamicin on viability of *P. aeruginosa* without (**a**) 1 M NaCl and (**b**) with 1 M NaCl.

We evaluated the activity of tobramycin, phosphomycin and gentamicin in the presence or absence of 1 M NaCl in order to determine whether the synergistic effect observed with IBU-Na was also present with other antibiotics commonly used in CF treatments. Figure 5a,b show that the presence of salt in a medium unlike that observed with IBU-Na does not increase the bactericidal effect of these antibiotics.

### 3.6. Langmuir Film Balance 

Considering that the presence of ionic strength modifies the activity of IBU-Na but not of other microbicides, we used tests in monomolecular layers to investigate whether the effect of salt was related to changes in the properties of surface activity of the IBU-Na. 

The adsorption of IBU-Na at the air-water interface was monitored by following the increase in surface pressure as a function of time. Gibbs adsorption isotherms are widely used in evaluating the extent of adsorption of surface-active compounds in dilute solutions. Another way to obtain IBU-Na film is to spread a solution at the air-water interface. Unlike monolayers resulting from adsorption which are in equilibrium with molecules in the bulk phase, spread monolayers are in a metastable state. In addition, with the aim of investigating the interactions between IBU-Na and cell membranes, the lipid monolayer is a very suitable model for study.

#### 3.6.1. Langmuir’s Monolayers

As previously reported by Jablonowska et al. in the absence of lipids, ibuprofen does not form a Lagmuir monolayer itself in aqueous subphase, independently of ionic strength (IS) (data not shown). 

#### 3.6.2. Gibbs Monolayers

The surface properties of IBU-Na at air-buffer interfaces were evaluated. The amphipathic compounds show a tendency to be located in an orderly manner at the interface, which is why they are said to be interfacially active materials. The surface activity of a compound is influenced, among others, by conformational factors, such as amphipathic structure, molecular size, molecular flexibility and net charge.

**Figure 6:**
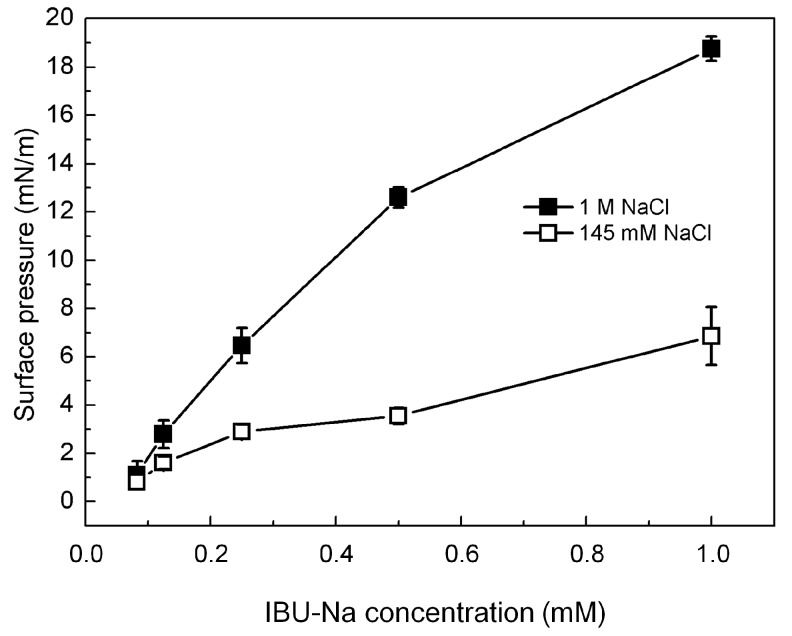
IBU-Na adsorption at air-buffer (100 mM TRIS-HCl buffer pH 7.6) interface. Maximum surface pressure versus IBU-Na concentration (mM) with 145 mM (empty squares) and 1 M NaCl (full squares).

Figure 6 shows that IBU-Na is able to absorb at the air-buffer interface with and without ionic strength, although IBU-Na reaches higher π with high ionic strength (1 M NaCl) at all concentrations evaluated, and the equilibrium surface pressure is more stable in 1 M NaCl condition (Figure 6), suggesting a better adsorption to the interface. 

**Figure 7:**
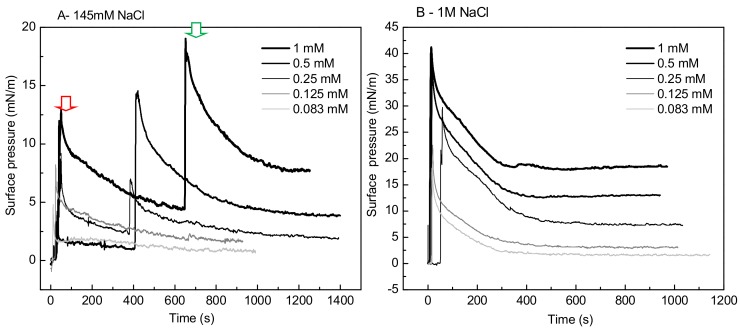
Effect of salt concentration on the absorption of IBU-Na. Surface pressure (at the air-buffer interface) versus time curves of increasing concentrations of IBU-Na with 145 mM (**A**) and 1 M NaCl (**B**).

A striking singular behavior is observed in the adsorption profile (π vs. time) of IBU-Na injected into subphase with low ionic strength (145 Mm NaCl). After the initial π peak due to the injection of the drug into the subphase (red arrow), a second pronounced (green arrow) peak is observed, after which π drops slowly to stabilize. This phenomenon is concentration-dependent, since it was only observed for concentrations of IBU-Na in subphase above 0.25 mM (Figure 7A).

The abrupt form in which the surface pressure increases, together with the fact that it occurs after a period in which π decreases slowly while there is a gradual accumulation of IBU in the subphase, suggests the participation of a self-aggregation phenomenon of the IBU-Na that depends on its concentration in the subphase. The progressive accumulation of IBU-Na in the subphase would probably favor the formation of a state of aggregation until reaching a critical point in which the abrupt increase of π would be driven.

#### 3.6.3. Penetration Studies

**Figure 8:**
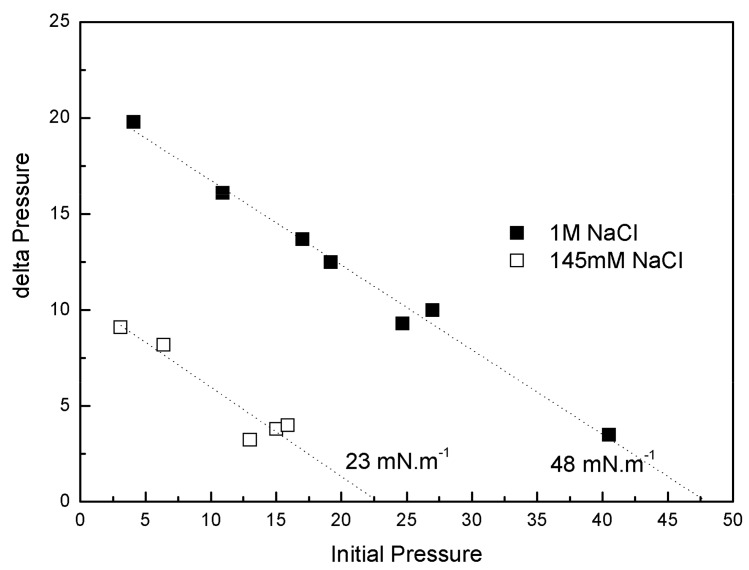
Penetration of IBU-Na into bacterial phospholipid monolayers. (∆π)_max_ of IBU-Na versus initial surface pressure of phospholipid monolayers with 145 mM (empty squares) and 1 M (full squares) NaCl.

IBU-Na was injected into a subphase of previously formed monolayers of phospholipids extracted from the Gram-negative bacilli *P. aeruginosa* (PM), with 145 mM and 1 M saline concentration. The increase of π caused by IBU-Na depended on the initial π of PM. For similar initial π the difference of π achieved was greater for higher ionic strength, suggesting that the presence of high salt concentration favors the penetration of IBU-Na into PM (Figure 8). This is also supported by the surface exclusion pressure, 23 mN·m^−1^ for subphases with 145 mM of and 48 mN·m^−1^ with 1 M of NaCl. This parameter is obtained by extrapolating the linear regression to a delta π equal to zero and reflecting the π of the lipid monolayer into which no more IBU-Na molecules would penetrate (Figure 8). 

**Figure 9:**
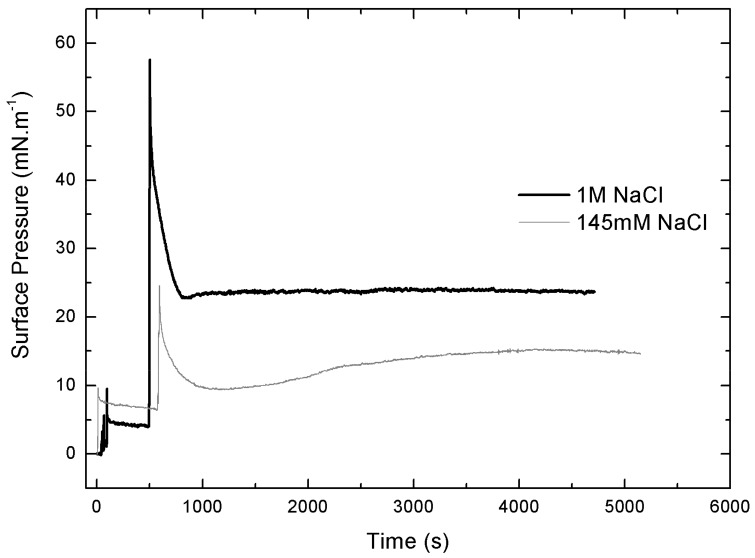
Effect of salt concentration on the penetration of IBU-Na. Surface pressure versus time of IBU-Na injected into subphases of bacterial phospholipids with 145 mM and 1 M NaCl.

The adsorption profiles (π vs. time) of IBU-Na injected into a subphase with a preformed lipid monolayer show a singular behavior, similar to the adsorption profiles. With greater subphase IS, the initial π of PLB monolayers is more stable, the equilibrium post-injection of Ibu is reached faster and constant over time. However, with low ionic strength π of PM decreases gradually and stabilizations of π after the injection of IBU-Na take more time (Figure 9). 

**Figure 10:**
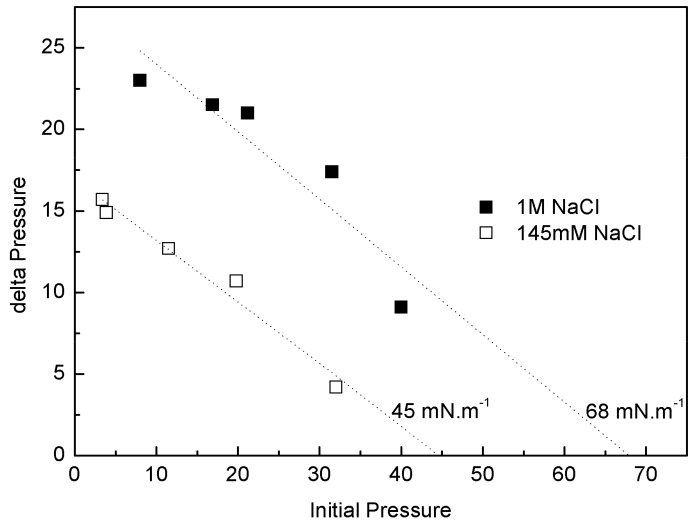
Penetration of IBU-Na into DOPE. (∆π)_max_ of IBU-Na versus initial surface pressure of DOPE monolayers with 145 mM (empty squares) and 1 M (full squares) NaCl.

The injection peak is followed by a progressive decrease of π and then an increase up to a π of equilibrium, suggesting a gradual rearrangement of the molecules at the interface (Figure 9). Moreover, with the initial π of 5 Mn·m^−1^, a second pressure peak is observed, similar to that seen in Gibbs monolayers (data not shown).As major phospholipids of bacterial wall are PE (neutral phospholipid with a cationic amino group at the end) and cardiolipin (negative phospholipid), we used monolayers of a pure lipid with positive charge, DOPE, to evaluate whether the observed interaction involved electrostatic interaction between IBU-Na and amino group of lipids. The effect of ionic strength on penetration of IBU-Na in DOPE monolayer was similar to PM, showing higher exclusion pressure with higher ionic strength (Figure 10).

### 3.7. Preclinical Toxicity Study

To evaluate the potential lung toxicity of IBU-Na in an inhalable form, experimental animals were exposed to inhalation of IBU-Na during 1 h a day for 4 months in two incremental doses (25 and 50 mM). 

No biochemical or behavioral changes were observed at the end of treatment. Histopathology of vital organs revealed no significant changes with respect to their weight or general morphology. Table 1 shows a summary of the findings found.

## 4. Discussion

In this report, we describe the synergic antibacterial activity of IBU in the presence of hypertonic solution of 1 M NaCl and consider its potential use for the treatment of bacterial agents involved in the development of pulmonary pathologies in fibrotic patients. 

The main contribution of this work concerns the fact that the combination of IBU with salts improves ten times its antimicrobial activity. In the treatment of cystic fibrosis, the use of concentrated solutions of salts (7% NaCl) is a common practice to achieve better mucus clearance from the lungs. Furthermore, this molecule does not interact with anionic polymers such as alginate or with DNA, major components of biofilms that hinder the access of cationic antibiotics such as Tobramycin.

Our results show that IBU in aqueous solution (IBU-Na) is effective at lower concentrations, as compared to IBU-H, and its activity is similar in the three pathogens evaluated. Regarding the activity of IBU-Na, its effect is observed in the pH range between 6.8 and 7.8. The loss in activity observed at pH below 6.8 can be explained by the shift from IBU-Na to IBU-H which produced a large decrease in drug solubility. On the other hand, a pH above 8 leads to a condition in which most *P. aeruginosa* cell cultures develop poorly.

The sensitizing effect of ionic strength, which only favors the action of IBU, is the most interesting finding among the results presented. This phenomenon can be caused by the decrease in the zeta potential with increasing ionic strength [23], showing that the variation of zeta potential is associated with increased permeability which, beyond a critical point, leads to cell death [24]. 

The fact that salt concentration must be present to promote the activity of IBU reinforces this point; a simple washing of bacterial suspension reverts its sensitivity to its baseline. On the other hand, the fact that the presence of salt produces a reduction in the minimum time required by IBU-Na for killing bacteria, from 120 min to only 2 min, strongly suggests that the effect of ionic strength would be related to the effect that IBU-Na produces on the stability of the membrane, rather than on an effect on metabolic pathways. It should be noted that, at this time, there is general consensus on the primary role of pulmonary inflammation in CF-associated pathology, in circumstances even dissociated from infection [25]. Neutrophils are also reported to be mainly responsible for this condition. Airway neutrophilia is associated with high levels of mediators that increase the migration of neutrophils to the lungs (interleukin-8, IL-8) [26] and lung tissue damage (neutrophil-derived elastase). Consequently, therapies with dual anti-infective and anti-inflammatory activity have been evaluated.

The treatments with anti-inflammatory molecules show that oral or inhaled corticosteroids are not recommended since they are associated with long-term side effects. However, studies with NSAIDs show that ibuprofen appears to be less toxic and more effective, even for long-term use in children [27,28,29,30]. In this report, we describe a formulation of IBU in high ionic strength that could improve CF treatments. IBU shows bactericidal activity against the three bacterial strains that mainly affect patients with CF, the presence of high ionic strength, a normal condition used in a nebulizable composition to fluidify and facilitate removal of the biofilm of lungs and increase bactericidal effect of IBU. This condition makes it possible to obtain a bactericidal effect at very low concentrations of IBU and, in particular, short periods of time, to which its anti-inflammatory effect could be added.

On the other hand, to gain insight into the effect of salt concentration on the activity of the IBU, we used defined interfacial systems such as Langmuir monolayers to investigate, at molecular level, [commas] the surface activity and interaction with lipid monolayers of IBU and how they change depending on whether 145 mM or 1 M NaCl is used. The results showed that although the IBU does not form Langmuir´s monolayers in the absence of lipids, it is adsorbed from the subphase to the air: buffer interface and this behavior is different according to the saline concentration of the subphase. The pressure reached by the molecule in the interface is higher and more stable with more amount of NaCl. The same was observed in penetration tests in lipid monolayers, where the insertion of the IBU is significantly greater in the presence of high ionic strength. Moreover, an unexpected effect of the ionic strength on IBU-Na surface activity was found. After an initial peak of insertion of IBU in air-buffer interface from subphase with 145 mM, when the samples were allowed to stand, a second and spontaneous peak of IBU pressure appears in the surface. This suggests the existence of aggregation equilibrium of IBU-Na in the subphase under these conditions of ionic strength. This phenomenon is not observed with 1 M NaCl, which is probably masked by the high ionic strength of the medium. These results correlate with those of microbicidal activity of the IBU, where we can infer that the high concentration of salts favors the approach and insertion of the compound in the membrane, triggering changes in electrical conductivity and its resulting instability.

Finally, we have also evaluated the potential toxicity of IBU-Na in lung, since there are no nebulizable commercial formulations of this NSAID. Animal studies were performed using IBU-Na alone, without 1 M NaCl. The results show that ibuprofen inhalation does not seem to produce substantial changes in the general state of the animals or significant histopathological alterations in the organs analyzed. Even though these studies are preliminary and there is still a lot to evaluate, the results obtained so far are particularly promising.

Complementary studies and evaluation of new protocols for obtaining animal models of lung infection are in progress to continue with the analysis of IBU/NaCl 1 M combination as a candidate treating patients with fibrosis. 

## Figures and Tables

**Table 1:** Histological analysis of rats´ lungs treated with IBU-Na.

TRT	Dose IBU [mM]	Acute Damage Parameters					Sub-Acute Damage Parameters	
		Alveolar Infiltrate	Interstitial Infiltrate	Hyaline Membranes	Protein Material	Septal Thickening	Masson Bodies	Granulomas and Giant Cells
A	25	0 ^a^	1	1	1	1	NO	NO
	50	0 ^b^	0 Capillary congestion	0	0	1 Low and limited	NO	NO
B	25	0	1	0	0	1	NO	NO
	50	0 ^b^	1	0	0	1	NO	NO
C	25	0 ^c^	1	0	0	1	NO	NO
	50	0 ^d^	1	0	0	1	NO	NO
D	25	0 ^c^	1	0	0	1	NO	NO
	50	0 ^d^	1	0	0	1	NO	NO

^a^ Alveolar hemorrhage; ^b^ Intense peribronchial mononuclear infiltrate; ^c^ Perivascular and peribronchiolar mononuclear infiltrate; ^d^ Mild peribronchial mononuclear infiltrate. A–B Group. Animals were nebulized with 25 and 50 mM IBU-Na 1 h a day for four months, C–D Group. Similar to A and B but after 4 months in each case they were left for 15 more days without any treatment and then sacrificed.
